# Morphometric Wing Characters as a Tool for Mosquito Identification

**DOI:** 10.1371/journal.pone.0161643

**Published:** 2016-08-23

**Authors:** André Barretto Bruno Wilke, Rafael de Oliveira Christe, Laura Cristina Multini, Paloma Oliveira Vidal, Ramon Wilk-da-Silva, Gabriela Cristina de Carvalho, Mauro Toledo Marrelli

**Affiliations:** 1 Departamento de Epidemiologia, Faculdade de Saúde Pública, Universidade de São Paulo, São Paulo, Brasil; 2 Instituto de Medicina Tropical de São Paulo, Universidade de São Paulo, São Paulo, Brasil; 3 Departamento de Parasitologia, Instituto Butantan, São Paulo, Brasil; Centro de Pesquisas René Rachou, BRAZIL

## Abstract

Mosquitoes are responsible for the transmission of important infectious diseases, causing millions of deaths every year and endangering approximately 3 billion people around the world. As such, precise identification of mosquito species is crucial for an understanding of epidemiological patterns of disease transmission. Currently, the most common method of mosquito identification relies on morphological taxonomic keys, which do not always distinguish cryptic species. However, wing geometric morphometrics is a promising tool for the identification of vector mosquitoes, sibling and cryptic species included. This study therefore sought to accurately identify mosquito species from the three most epidemiologically important mosquito genera using wing morphometrics. Twelve mosquito species from three epidemiologically important genera (*Aedes*, *Anopheles* and *Culex*) were collected and identified by taxonomic keys. Next, the right wing of each adult female mosquito was removed and photographed, and the coordinates of eighteen digitized landmarks at the intersections of wing veins were collected. The allometric influence was assessed, and canonical variate analysis and thin-plate splines were used for species identification. Cross-validated reclassification tests were performed for each individual, and a Neighbor Joining tree was constructed to illustrate species segregation patterns. The analyses were carried out and the graphs plotted with TpsUtil 1.29, TpsRelw 1.39, MorphoJ 1.02 and Past 2.17c. Canonical variate analysis for *Aedes*, *Anopheles* and *Culex* genera showed three clear clusters in morphospace, correctly distinguishing the three mosquito genera, and pairwise cross-validated reclassification resulted in at least 99% accuracy; subgenera were also identified correctly with a mean accuracy of 96%, and in 88 of the 132 possible comparisons, species were identified with 100% accuracy after the data was subjected to reclassification. Our results showed that *Aedes*, *Culex* and *Anopheles* were correctly distinguished by wing shape. For the lower hierarchical levels (subgenera and species), wing geometric morphometrics was also efficient, resulting in high reclassification scores.

## Introduction

### Mosquito importance

Insects that are obligate blood feeders, such as the Culicidae family, can have a major impact on human and animal health, primarily because they transmit infectious agents, and their bites constitute a significant inconvenience. The more urbanized an area is, the lower the mosquito richness and the higher the abundance of a few, epidemiologically important mosquito species that are well adapted to urban environments [1–3]. Mosquitoes of the *Aedes*, *Culex* and *Anopheles* genera are responsible for the transmission of highly important infectious diseases such as dengue, filariasis, West Nile virus disease and malaria. Together, these diseases cause millions of deaths every year and endanger approximately 3 billion people around the world living in endemic areas. Expansion of the geographic distribution of mosquitoes is followed by the (re-)emergence of diseases, making it vital to identify epidemiologically important mosquito species correctly [4–18].

Precise species identification of mosquitoes is crucial for an understanding of epidemiological patterns of disease transmission, which are associated with the vector mosquito’s abundance, infectivity and vector capacity and competence. In addition, morphologically indistinguishable species complexes such as the *An*. *gambiae* and *Cx*. *pipiens* complexes may have distinct epidemiological roles, making correct identification of the species in these complexes crucial to establish an effective vector mosquito control initiative [19–23].

The most common method of mosquito identification relies on the use of morphological taxonomic keys [14,24–26], a laborious process that requires intensive training and, most importantly, that the mosquito be undamaged. If for any reason the mosquito specimen of interest is damaged, morphological identification may not be possible. Moreover, some species can only be identified by quantitative differences in the male genitalia, making it impossible to identify adult females, or may even be morphologically indistinguishable (e.g. *An*. *gambiae* and *Cx*. *pipiens* complex) [19,27]. The vector species *An*. *cruzii*, *An*. *homunculus* and *An*. *bellator*, considered difficult to identify using only taxonomic keys, have been successfully identified based only on adult female wing morphometry [28]. Jaramillo-O et al. [29] were able to distinguish eleven *Anopheles* mosquito species from the *Nyssorhynchus* subgenus using wing geometric morphometrics.

Mosquito wing geometric morphometrics is an established mosquito identification technique and is inexpensive and reliable [30–33]. It can be used to identify vector mosquitoes of epidemiological importance, sibling species, cryptic species and females in some species whose identification using other techniques has proved problematic [20,23,28]. The objective of this study was to accurately identify mosquito species from the three main epidemiologically important mosquito genera using wing morphometrics.

## Material and Methods

### Mosquito sampling and identification

Twelve species of mosquitoes from the three most epidemiologically important genera (*Ae*. *aegypti*, *Ae*. *albopictus*, *Ae*. *fluviatilis*, *Ae*. *scapularis*, *An*. *cruzii*, *An*. *darlingi*, *An*. *strodei*, *Cx*. *chidesteri*, *Cx*. *dolosus*, *Cx*. *eduardoi*, *Cx*. *nigripalpus* and *Cx*. *quinquefasciatus*) were collected from different sampling locations (Table 1) as previously described by Medeiros-Sousa et al. [2]. Adults were collected using CO_2_-baited CDC light traps, and immature forms were collected in natural and artificial breeding sites with larval dippers or suction tubes. The mosquitoes were identified with the aid of taxonomic keys [24] and stored by species in 1.5 mL Eppendorf tubes with silica gel at room temperature until the wings were removed. The study was approved by the Ethical Committee of the University of São Paulo (FSP/USP—Project 000304), and collection permits were provided by the Department of the Environment and Green Areas (Permit 345/2010).

**Table 1:** Mosquito species, collection sites and geographic coordinates.

Taxon	N*	Collection site	Coordinate	Collection year
*Aedes* (*Stegomyia*) *aegypti* (Linnaeus, 1762)	28	São Paulo	S-23°24′54”, W-46°47′6”	2013
*Aedes* (*Stegomyia) albopictus* (Skuse, 1895)	23	São Paulo	S-23°31’40” W-46°34’14”	2013
*Aedes* (*Ochlerotatus*) *fluviatilis* (Lutz, 1904)	30	São Paulo	S-23°34’49” W-46°43’33”	2013
*Aedes* (*Ochlerotatus*) *scapularis* (Rondani, 1848)	23	São Paulo	S-23°31’40” W-46°34’14”	2013
*Anopheles* (*Kerteszia*) *cruzii*	22	São Paulo	S-23°82'69'' W-46°72'70''	2015
*Anopheles* (*Nyssorhynchus*) *darlingi*	30	Manaus	S-3°11'89'' W-60°02'15''	2015
*Anopheles* (*Nyssorhynchus*) *strodei* Root, 1926	28	São Paulo	S-23°24′54”, W-46°47′6”	2013
*Culex* (*Culex*) *chidesteri* Dyar, 1921	28	São Paulo	S-23°31’40” W-46°34’14”	2012
*Culex* (*Culex*) *dolosus* (Lynch Arribálzaga, 1891)	29	São Paulo	S-23°34’40” W-46°43’37”	2012
*Culex* (*Culex*) *eduardoi* Casal & García, 1968	14	São Paulo	S-23°45’29” W-46°46’23”	2013
*Culex* (*Culex*) *nigripalpus* Theobald, 1901	29	São Paulo	S-23°49'25'' W-46°76'17''	2013
*Culex* (*Culex*) *quinquefasciatus* Say, 1823	28	São Paulo	S- 23°37’55” W-46°43’17”	2013

*Number of specimens used

### Material preparation and data acquisition

The right wing of each adult female mosquito was removed and mounted on a microscope slide with a cover slip. The wings were then photographed under 40x magnification with a Leica DFC320 digital camera coupled to a Leica S6 microscope. On each wing image, 18 landmarks were digitized by one of the authors (ROC) using TpsDig V1.40 software (S1 Fig) [28,30,34,35].

### Geometric morphometric analysis

The allometric influence of wing size on wing shape was assessed by multivariate regression of the Procrustes coordinates against centroid size using a permutation test with 10000 randomizations. Discriminant analysis was performed to explore the degree of wing shape dissimilarity among species in a morphospace produced by canonical variate analysis (CVA) and to calculate the Mahalanobis distance. Thin-plate splines were obtained by regression analysis of CVA scores against wing shape variation to visualize the shape disparity among the species compared. Each individual was then reclassified using the cross-validated reclassification test based on the Mahalanobis distance [28,36,37]. A Neighbor Joining tree was constructed with 1000 bootstrap replicates based on the Mahalanobis distance to illustrate species segregation patterns (20 specimens of *Wyeomyia oblita*, were used as an outgroup). The analyses were carried out and graphs plotted with TpsUtil 1.29 [38], TpsRelw 1.39 [38], MorphoJ 1.02 [39] and Past 2.17c [40].

## Results

**Fig 1:**
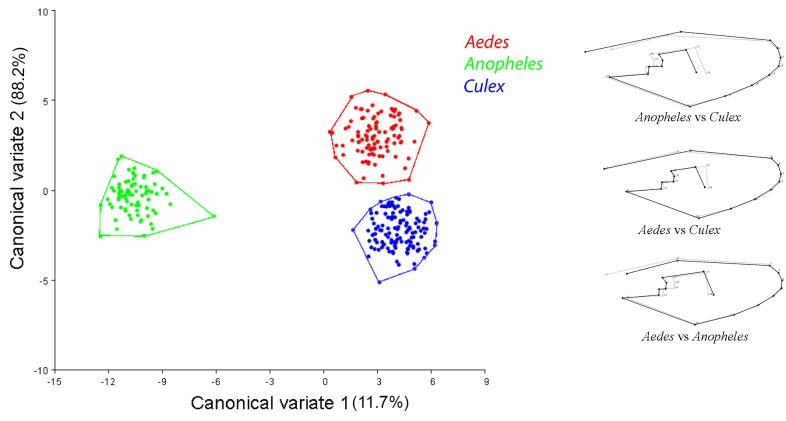
Morphological space produced by CVA of the three mosquito genera. Shape variations in a wireframe graph are shown to the right of the morphological space.

Although small, the allometry effect was significant (5.91%, p<0.0001). However, it was not removed from the analysis because we consider allometric size variation part of the species identification process [37]. CVA of wing shape in *Aedes*, *Anopheles* and *Culex* genera showed three distinct clusters in morphospace and correctly distinguished between the three mosquito genera. Subsequent pairwise comparison of thin-plate splines between genera showed that quantitative landmarks are associated with differentiation among mosquitoes from each genus (Fig 1, S2 Fig). A pairwise cross-validated reclassification test comparing the three genera (*Aedes*, *Anopheles* and *Culex*) was also performed and resulted in scores with an accuracy of at least 99%.

**Fig 2:**
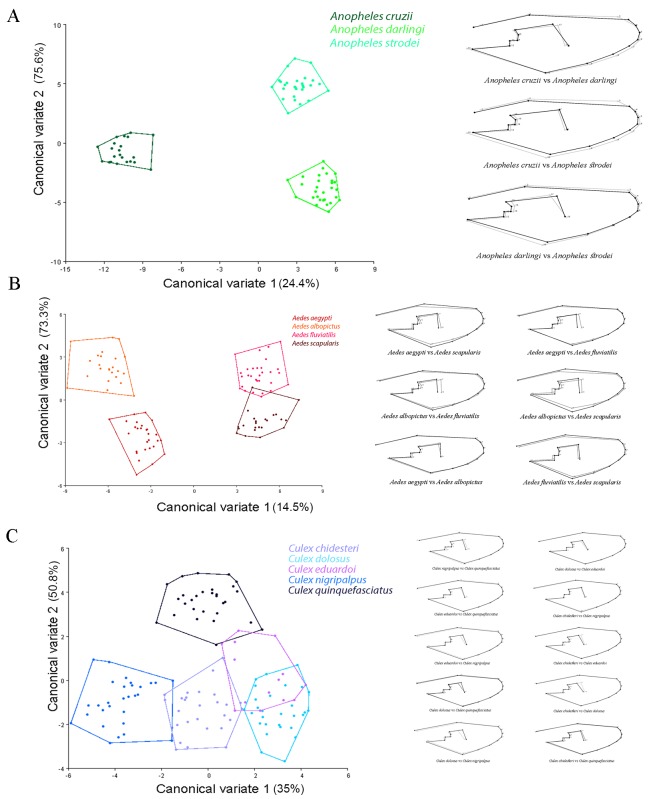
Morphological space produced by CVA of species and shape variations in a wireframe graph. **A.** *Anopheles* genus. **B.** *Aedes* genus. **C.** *Culex* genus. Shape variations in a wireframe graph are shown to the right of each morphological space.

The results obtained within each genus show that subgenera were identified when present. The *Anopheles* genus was correctly segregated into two main clusters, the *Kerteszia* and *Nyssorhynchus* subgenera (Fig 2A), and the *Aedes* genus was also divided into two clusters, the *Stegomyia* and *Ochlerotatus* subgenera (Fig 2B).

**Fig 3:**
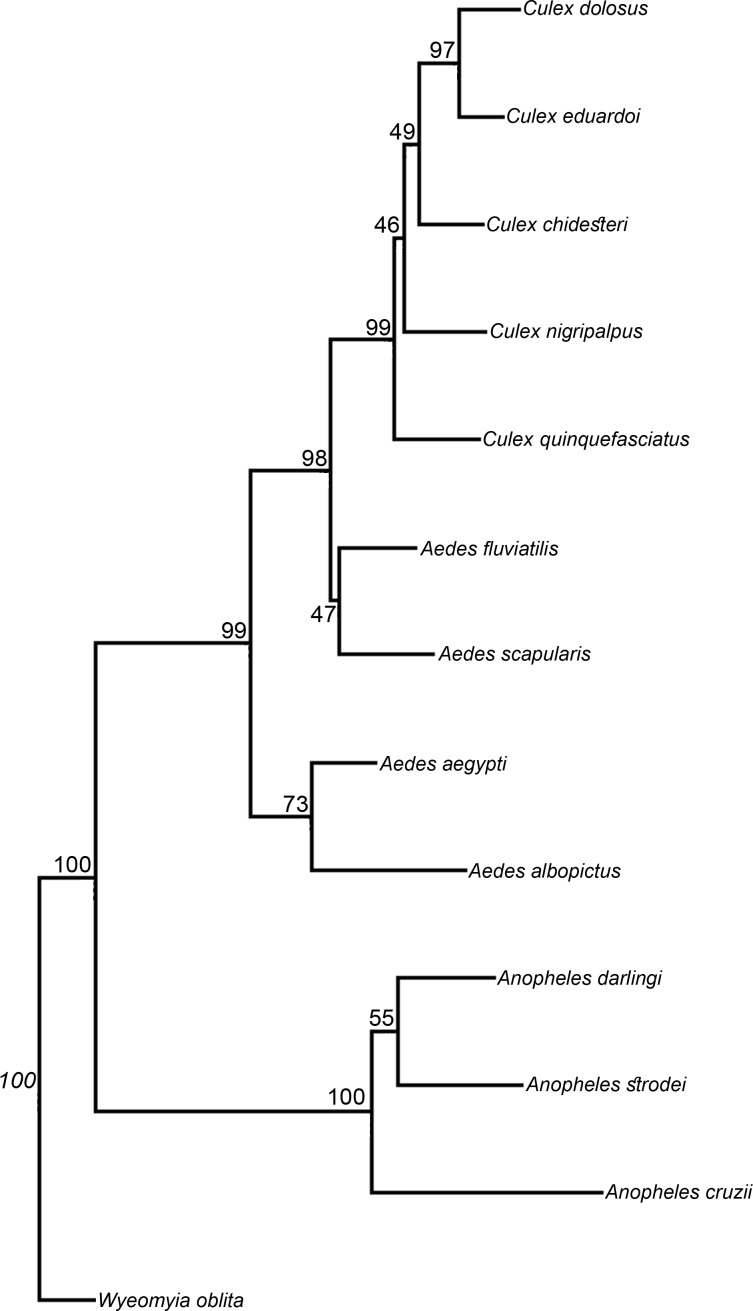
Neighbor-Joining tree based on Mahalanobis distance with 1000 bootstrap replicates.

CVA was carried out among the species of each genus followed by a thin-plate spline pairwise comparison and yielded the following results: in the genus ***Anopheles***, wing shape CVA successfully separated the species (*An*. *cruzii*, *An*. *darlingi* and *An*. *strodei*), and the subgenera *Kerteszia* and *Nyssorhynchus* segregated perfectly along the canonical variate 1 (CV1) axis (Fig 2A). In the genus ***Aedes***, *Ae*. *aegypti*, *Ae*. *albopictus*, *Ae*. *fluviatilis* and *Ae*. *scapularis* were successfully separated, and the subgenera *Stegomyia* and *Ochlerotatus* (Fig 2B) segregated with a small degree of overlapping along the CV1 axis. In the genus ***Culex***, wing-shape CVA revealed structuring within the genus; *Cx*. *nigripalpus* was the most segregated species, and *Cx*. *dolosus* and *Cx*. *eduardoi* overlapped (Fig 2C). The Neighbor Joining tree shows the segregation of the three mosquito genera, subgenera and respective species with high bootstrap values in most branches (Fig 3).

The genera were correctly identified by morphometric analysis with at least 99% reliability after a reclassification test, indicating a high reliability rate. The reclassification test also had a high identification power when each genera was analyzed separately and was able to distinguish subgenera correctly when these were present.

Mean species-identification reliability after the data were subjected to a reclassification test was 96%, and in 88 out of 132 comparisons accuracy was 100%. Comparisons of the results of pairwise cross-validated species reclassification tests between species (*Ae*. *aegypti*, *Ae*. *albopictus*, *Ae*. *fluviatilis*, *Ae*. *scapularis*, *An*. *cruzii*, *An*. *darlingi*, *An*. *strodei*, *Cx*. *chidesteri*, *Cx*. *dolosus*, *Cx*. *eduardoi*, *Cx*. *nigripalpus* and *Cx*. *quinquefasciatus*) yielded high identification rates in the majority of the comparisons. *Ae*. *aegypti*, *An*. *darlingi*, *An*. *strodei* and *Cx*. *quinquefasciatus* had a reclassification score of 100% when they were compared with mosquito species from different genera. When *Cx*. *eduardoi* was compared with *Cx*. *dolosus*, its reclassification score was only 57%, the lowest score in all the comparisons of species carried out, although its mean reclassification score was 80%. The overall mean reclassification score for both groups 1 and 2 in the pairwise cross-validated reclassification tests was 96%, indicating a high species-identification reliability (Table 2).

**Table 2:** Results of pairwise cross-validated species reclassification tests.

		Group 2											
		*Ae*. *aegypti*	*Ae*. *albopictus*	*Ae*. *fluviatilis*	*Ae*. *scapularis*	*An*. *cruzii*	*An*. *darlingi*	*An*. *strodei*	*Cx*. *chidesteri*	*Cx*. *dolosus*	*Cx*. *eduardoi*	*Cx*. *nigripalpus*	*Cx*. *quinquefasciatus*
Group 1	*Ae*. *aegypti*	x	85%	100%	96%	100%	100%	100%	100%	100%	96%	100%	100%
	*Ae*. *albopictus*	83%	x	100%	95%	100%	100%	100%	100%	100%	95%	100%	100%
	*Ae*. *fluviatilis*	100%	100%	x	86%	100%	100%	100%	93%	96%	93%	93%	100%
	*Ae*. *scapularis*	95%	100%	91%	x	100%	100%	100%	95%	100%	82%	95%	100%
	*An*. *cruzii*	100%	100%	100%	100%	x	100%	100%	100%	100%	100%	100%	100%
	*An*. *darlingi*	100%	100%	100%	100%	100%	x	100%	100%	100%	96%	100%	100%
	*An*. *strodei*	100%	100%	100%	100%	100%	100%	x	100%	100%	96%	100%	100%
	*Cx*. *chidesteri*	100%	100%	96%	100%	100%	100%	100%	x	92%	85%	85%	96%
	*Cx*. *dolosus*	100%	100%	100%	100%	100%	100%	100%	96%	x	68%	100%	96%
	*Cx*. *eduardoi*	100%	85%	78%	64%	78%	100%	100%	85%	57%	x	85%	71%
	*Cx*. *nigripalpus*	100%	100%	96%	96%	100%	100%	100%	82%	93%	82%	x	100%
	*Cx*. *quinquefasciatus*	100%	100%	96%	93%	100%	100%	100%	89%	93%	82%	100%	x

Values below the diagonal correspond to mosquitoes from group 1 compared with group 2 and correctly identified; values above the diagonal correspond to mosquitoes from group 2 compared with group 1 and correctly identified. P-value (parametric): <0.0001.

The minimum number of landmarks required to distinguish genera with more than 90% accuracy in the cross-validated reclassification test was five (1, 2, 14, 15, 16). Using seven landmarks (1, 2, 14, 15, 16, 17, 18), 96% accuracy was achieved, and with nine (1, 2, 12, 13, 14, 15, 16, 17, 18) the lowest score was 97% (S3 Fig, S1 Table).

## Discussion

Although morphological analysis of characters is the gold standard for mosquito identification, geometric morphometrics based on quantitative analysis of mosquito wing venation characters has proved to be a reliable tool in mosquito identification as well, showing a high identification power when distinguishing morphologically similar species [28,41].

Our results indicate that the three most epidemiologically important mosquito genera, *Aedes*, *Culex* and *Anopheles*, were correctly distinguished by wing shape. When lower hierarchical levels (subgenera and species) were analyzed, wing geometric morphometrics was also efficient, yielding high reclassification scores for most of the mosquito species analyzed. In light of this, the technique can be considered a valid alternative for identification of the twelve mosquito species studied here when identification by taxonomic methods is not possible.

The morphospace pattern formed by the four *Aedes* species allows identification of the subgenus *Stegomyia* and the species within this subgenus in this study, i.e., *Ae*. *albopictus* and *Ae*. *aegypti*, the two most important vectors of dengue globally, although in Latin America the role of *Ae*. *albopictus* in dengue transmission is still unknown. Subgenus *Ochlerotatus*, represented by two species native to Brazil, was also successfully identified. Hence, identification of species of secondary epidemiological importance, about which less information is available, is also possible with the geometric morphometrics used here [42].

The *Anopheles* species used in this study are epidemiologically very important because they are responsible for malaria transmission in Latin America. *An*. *darlingi* is the primary vector of this disease in the Amazon region, and *An*. *cruzii and An*. *strodei* are responsible for malaria transmission in the Atlantic Forest region, accounting for hundreds of thousands of malaria cases every year [15,17].

Our results showed that wing geometric morphometrics is a reliable technique for identification of the *Anopheles* species used in this study at the genus, subgenus and species level. The three species tested formed a clear pattern in morphospace and exhibited significant morphometric singularities. Wing morphometrics was previously used by Lorenz et al. [28], who were able to identify the species *An*. *cruzii*, *An*. *homunculus* and *An*. *bellator* using wing morphometry. These species can usually only be identified by examination of male genitalia, making identification of females problematic.

Species from the *Culex* genus may be undergoing microevolution and speciation [43], leading to species complexes and even hybrid mosquitoes as a result of the mating of sibling species [41,44]. The species complex formed by *Cx*. *quinquefasciatus* and *Cx*. *pipiens*, which are adapted to tropical and temperate climates, respectively, is found in an overlapping tropical/temperate climate zone in Brazil, where the two species can mate, producing hybrid mosquitoes.

This phenomenon has been observed using two different approaches: wing morphometrics and microsatellite markers [41,44]. A similar finding was reported for *Cx*. *pipiens* and *Cx*. *torrentium*, which are only distinguishable by the characteristics of their male genitalia; although not even molecular tools such as COI barcodes are sufficiently sensitive to identify specific *Culex* species, wing morphometrics has been successfully used to identify species in this genus [20,45].

In the present study, we were able to correctly identify epidemiologically important *Culex* species, including *Cx*. *quinquefasciatus*, one of the main vectors of West Nile virus in the Americas [46], *Cx*. *nigripalpus*, a neglected mosquito native to South America that is very abundant in the urban environment [43,47], and *Cx*. *chidesteri*, which is also native to South America but less adapted to urban environments. As expected, *Cx*. *dolosus* and *Cx*. *eduardoi* overlapped in the CVA analysis and had the lowest reclassification scores. However, these species can only be identified by morphological differences in larval stages, and adults are indistinguishable.

The Neighbor Joining tree produced here corroborated the results of the wing shape CVA and reclassification test, showing that wing morphometrics clearly identified genus, subgenus and species and yielded results comparable to those obtained with classic morphological analysis [43,48–50].

The use of wing morphometrics for mosquito identification has a number of advantages over classical species identification based on morphological traits and genetic characterization. One such advantage is that wing characters respond rapidly to microevolutionary events. Thus, small but significant variations in sibling species or even hybrid mosquitoes can be identified, allowing them to be correctly identified [20,21,32,41,51,52].

Furthermore, variation in wing shape among species has proven to be a reliable marker that is consistent regardless of the geographic origin of the mosquitoes and does not produce significant interspecific overlapping in morphospace [32].

The use of wing morphometric characters can be an important tool in the identification of mosquito species that are not easily identified by morphological characters or specimens that are damaged during collection, making morphological identification unfeasible. There are freely accessible databases, such as the CLIC morphometrics database (mpl.ird.fr/morphometrics/clic/index.html) and WINGBANK (winngbank.com.br), that researchers can use to obtain photos of wings of different mosquito species for use as reference and for further analysis.

As long as one wing is preserved, specimens can be identified by morphometric techniques. These techniques are fast and relatively low-cost, especially compared with genetic characterization, and can be performed in the field without advanced equipment.

## Conclusion

We were able to successfully identify all the species analyzed in this study, including even cryptic species that cannot be identified by morphological characters or species whose males can be identified by their genitalia but whose females are indistinguishable. These findings suggest that wing geometric morphometrics is a reliable tool for mosquito identification.

## Supporting Information

S1 Fig Wireframe showing the 18 landmarks on wings of female *Aedes*, *Anopheles* and *Culex* mosquitoes selected for geometric morphometrics analysis. (TIF)

S2 Fig Morphological space produced by CVA of *Ae*. *aegypti*, *Ae*. *albopictus*, *Ae*. *fluviatilis*, *Ae*. *scapularis*, *An*. *cruzii*, *An*. *darlingi*, *An*. *strodei*, *Cx*. *chidesteri*, *Cx*. *dolosus*, *Cx*. *eduardoi*, *Cx*. *nigripalpus* and *Cx*. *quinquefasciatus*. (TIF)

S3 Fig Morphologicalspace produced by CVA of the three mosquito genera using **A.** Five landmarks (1, 2, 14, 15, 16); **B.** Seven landmarks (1, 2, 14, 15, 16, 17, 18); and **C.** Nine landmarks (1, 2, 12, 13, 14, 15, 16, 17, 18).
(TIF)

S1 Table Comparisons of the three genera (*Aedes*, *Anopheles* and *Culex*) using the pairwise cross-validated reclassification test and five, seven and nine landmarks. (DOCX)
