# Supplementary figures and images for: Morphometric Wing Characters as a Tool for Mosquito Identification
Source: PLoS One. 2016 Aug 23;11(8):e0161643. doi: 10.1371/journal.pone.0161643 (PMC4995034; doi:10.1371/journal.pone.0161643)

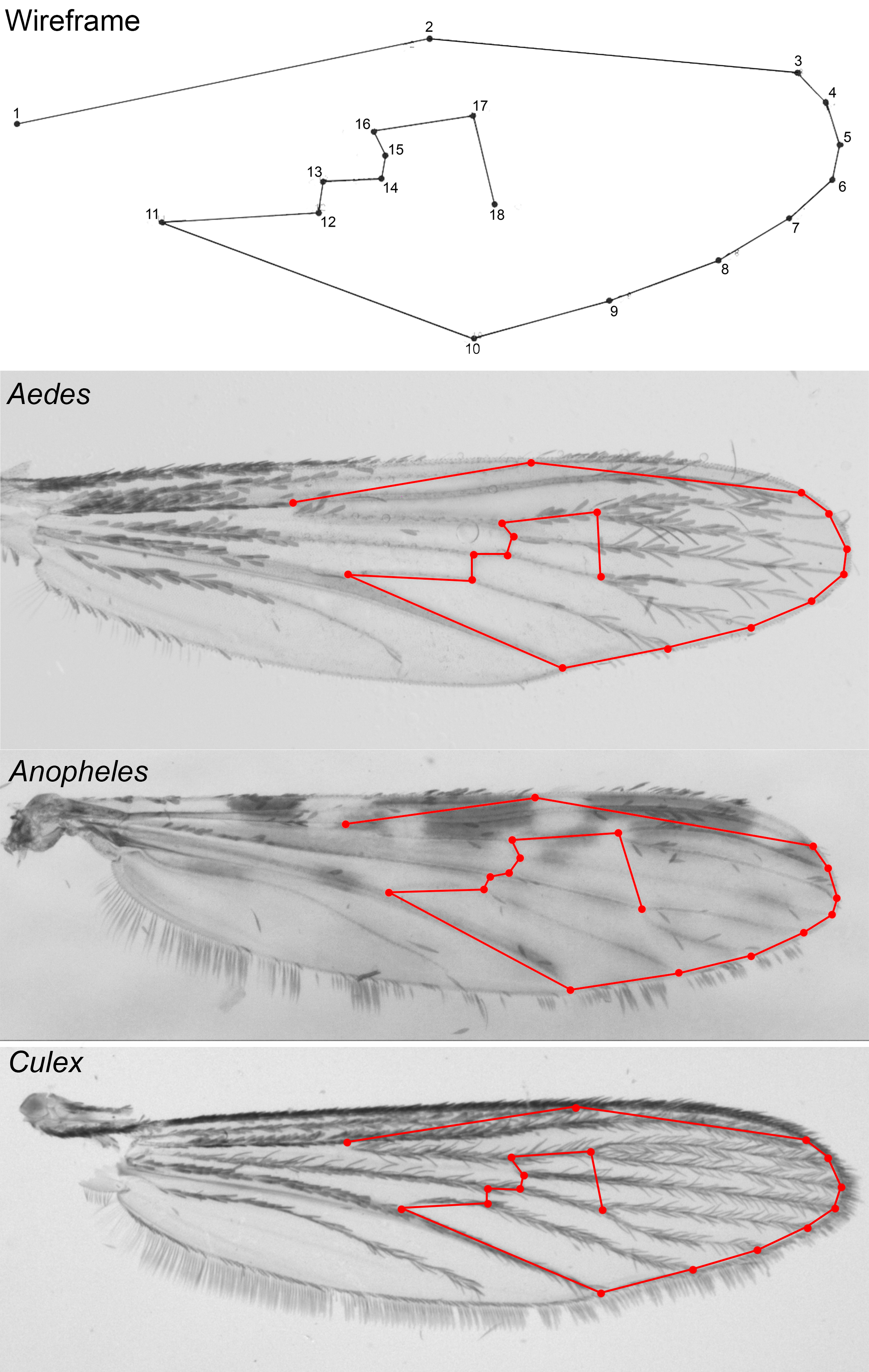

Supplement: S1 Fig — (TIF) [file pone.0161643.s001.tif]

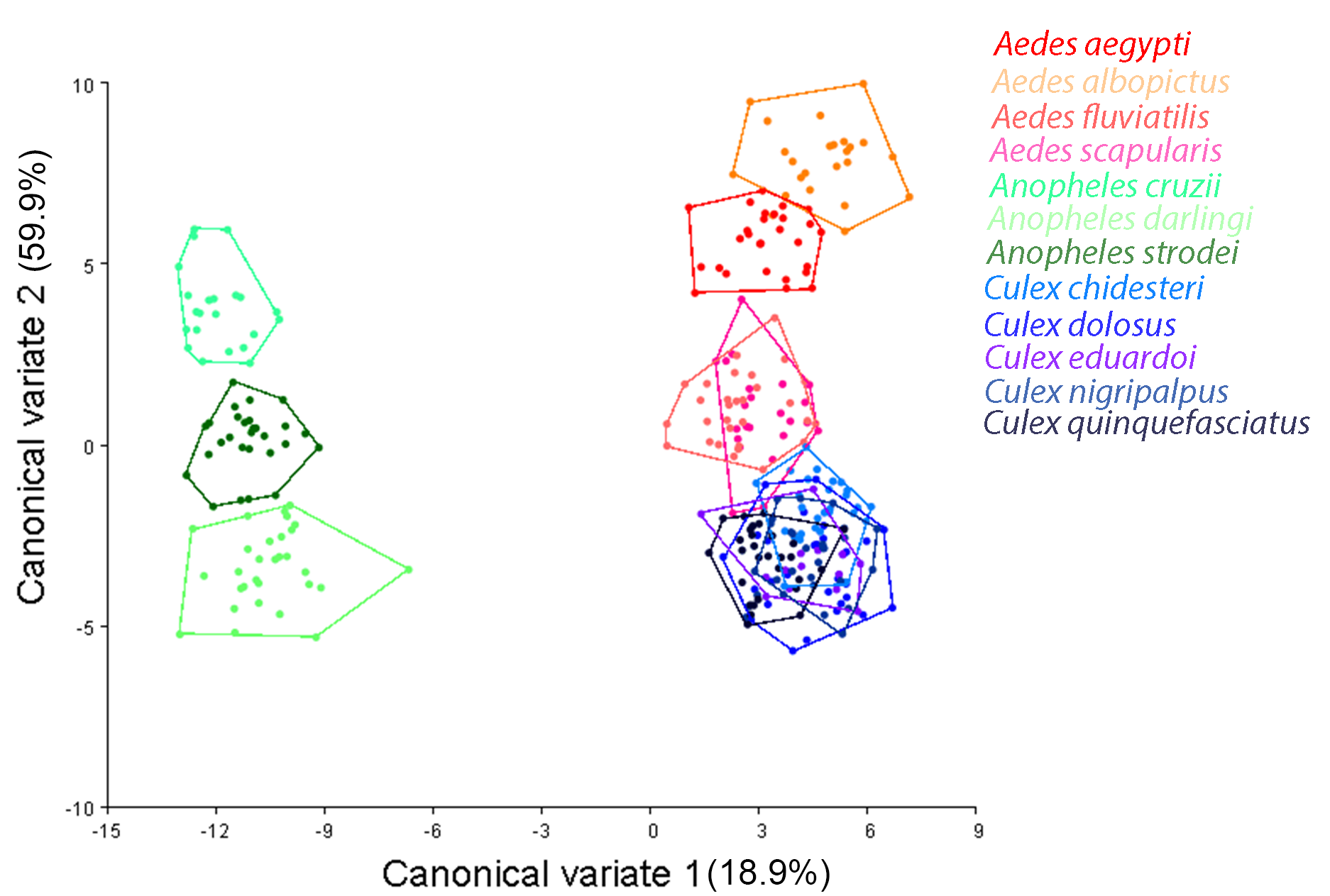

Supplement: S2 Fig — (TIF) [file pone.0161643.s002.tif]

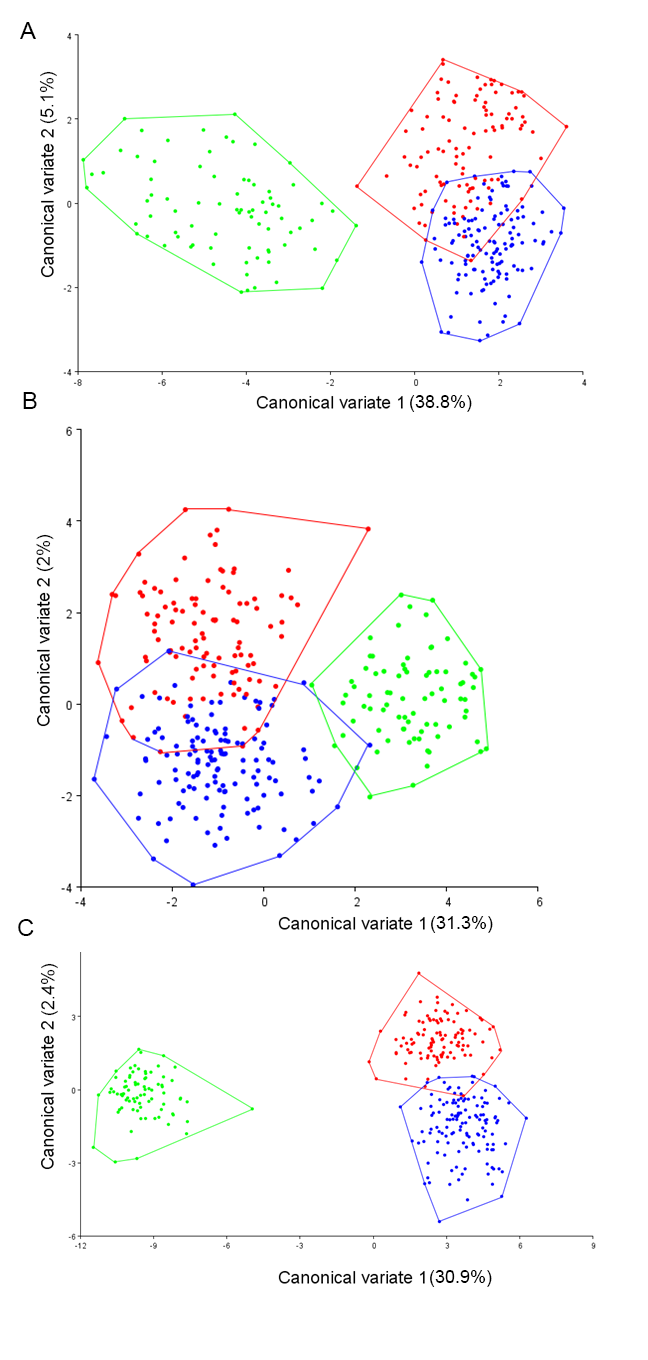

Supplement: S3 Fig — Morphologicalspace produced by CVA of the three mosquito genera using A. Five landmarks (1, 2, 14, 15, 16); B. Seven landmarks (1, 2, 14, 15, 16, 17, 18); and C. Nine landmarks (1, 2, 12, 13, 14, 15, 16, 17, 18). (TIF) [file pone.0161643.s003.tif]
