# Supplementary material for: Morphometric Wing Characters as a Tool for Mosquito Identification
Source: PLoS One. 2016 Aug 23;11(8):e0161643. doi: 10.1371/journal.pone.0161643 (PMC4995034; doi:10.1371/journal.pone.0161643)
Supplement: S1 Table — (DOCX) [file pone.0161643.s004.docx]

**Table S1.** Comparisons between the three genera (*Aedes*, *Anopheles* and *Culex*) using the pairwise cross-validated reclassification test and landmarks 5, 7 and 9.

| 5 landmarks (1, 2, 14, 15, 16) | | | | |  | 7 landmarks (1, 2, 14, 15, 16, 17, 18). | | | | |  | 9 landmarks (1, 2, 12, 13, 14, 15, 16, 17, 18) | | | | |
| --- | --- | --- | --- | --- | --- | --- | --- | --- | --- | --- | --- | --- | --- | --- | --- | --- |
|  |  | Group 2 | | |  |  |  | Group 2 | | |  |  |  | Group 2 | | |
|  |  | *Aedes* | *Anopheles* | *Culex* |  |  |  | *Aedes* | *Anopheles* | *Culex* |  |  |  | *Aedes* | *Anopheles* | *Culex* |
| Group 1 | *Aedes* | x | 100% | 93% |  | Group 1 | *Aedes* | x | 100% | 98% |  | Group 1 | *Aedes* | x | 100% | 98% |
|  | *Anopheles* | 100% | X | 100% |  |  | *Anopheles* | 100% | X | 100% |  |  | *Anopheles* | 100% | X | 100% |
|  | *Culex* | 90% | 100% | x |  |  | *Culex* | 96% | 100% | x |  |  | *Culex* | 97% | 100% | x |

Percentage values for pairwise cross-validated reclassification: values below the diagonal line are for mosquitoes from group 1 compared with group 2 and correctly identified. Values above the diagonal line are for mosquitoes from group 2 compared with group 1 and correctly identified. P-value (parametric) <0.0001.
